# Response of cytokinins and nitrogen metabolism in the fronds of *Pteris* sp. under arsenic stress

**DOI:** 10.1371/journal.pone.0233055

**Published:** 2020-05-15

**Authors:** Daniela Pavlíková, Veronika Zemanová, Milan Pavlík, Petre I. Dobrev, František Hnilička, Václav Motyka

**Affiliations:** 1 Department of Agro-Environmental Chemistry and Plant Nutrition, Faculty of Agrobiology, Food and Natural Resources, Czech University of Life Sciences Prague, Prague, Czech Republic; 2 Isotope Laboratory, Institute of Experimental Botany of the Czech Academy of Sciences, Prague, Czech Republic; 3 Laboratory of Hormonal Regulations in Plants, Institute of Experimental Botany of the Czech Academy of Sciences, Prague, Czech Republic; 4 Department of Botany and Plant Physiology, Faculty of Agrobiology, Food and Natural Resources, Czech University of Life Sciences Prague, Prague, Czech Republic; RIKEN Biomass Engineering Program, JAPAN

## Abstract

Given the close relationship between cytokinins (CKs), photosynthesis and nitrogen metabolism, this study assessed the effect of arsenic (As) contamination on these metabolic components in the As-hyperaccumulators *Pteris cretica* L. var. Albo-lineata (*Pc*-A) and var. Parkerii (*Pc*-P) as well as the As-non-hyperaccumulator *Pteris straminea* Mett. ex Baker (*Ps*). The ferns were cultivated in a pot experiment for 23 weeks in soil spiked with As at the levels 20 and 100 mg·kg^-1^. For the purpose of this study, the CKs were placed into five functionally different groups according to their structure and physiological roles: bioactive forms (bCKs; CK free bases); inactive or weakly active forms (dCKs; CK *N*-glucosides); transport forms (tCKs; CK ribosides); storage forms (sCKs; *O-*glucosides); and primary products of CK biosynthesis (ppbCKs; CK nucleotides). An important finding was higher CKs total content, accumulation of sCKs and reduction of dCKs in As-hyperaccumulators in contrast to non-hyperaccumulator ferns. A significant depletion of C resources was confirmed in ferns, especially *Ps*, which was determined by measuring the photosynthetic rate and chlorophyll fluorescence. A fluorescence decrease signified a reduction in the C/N ratio, inducing an increase of bioactive CKs forms in *Pc*-P and *Ps*. The impact of As on N utilization was significant in As-hyperaccumulators. The glutamic acid/glutamine ratio, an indicator of primary N assimilation, diminished in all ferns with increased As level in the soil. In conclusion, the results indicate a large phenotypic diversity of *Pteris* species to As and suggest that the CKs composition and the glutamic acid/glutamine ratio can be used as a tool to diagnose As stress in plants.

## Introduction

Cytokinins (CKs), together with other phytohormones, play a crucial role in the ability of plants to adapt to changing environments by mediating their growth and development. They have extensive biological effects on plants, such as stimulating cell division, promoting plant cotyledon expansion, delaying leaf senescence, promoting adaptation to stress and facilitating an antioxidative plant system and chlorophyll biosynthesis [1,2]. Metabolic and physiological processes in plants are regulated by hormone homeostasis. Cytokinins coordinate these processes in concert with phytohormones (auxins, abscisic acid and ethylene) [3–8], above all by regulating cytokinin homeostasis via five functionally different CK groups divided in terms of biological function/structure [9–12].

The bioactive forms (bCKs; CK free bases) are regulated by changes in the synthesis of free CKs. Free bCKs delay senescence, increase photosynthesis, increase/decrease nutrient uptake via the roots through regulating both root and aboveground growth and development, flowers and seed development and additionally affect the biosynthesis of antioxidant metabolites such as flavonoids, purple anthocyanins and lignins [10,13,14]. The bioactive forms are primarily synthesized in the roots and transported by transport forms of CKs (tCKs; riboside conjugates with weak activity). Irreversible bCK degradation is catalyzed by cytokinin oxidase/dehydrogenase (CKX), resulting in the formation of adenine or adenosine and 3-methyl-2-butenal (isoprene aldehyde—C5) [15,16]. Other important CK forms include two conjugated forms that differ in biological function/structure and reversible/irreversible degradation capacity. Store/conjugate *O*-glucosyl CK forms (sCKs) are inactive storage forms which are not degradable by CKX. They can be hydrolyzed by β-glucosidase and converted from sCKs to free bCKs. The accumulation of sCKs is associated with senescence and stress [9,10,17,18]. Terminal deactivated/conjugate *N*-glucoside CK forms (dCKs) are associated with the flowering period, with the end of the life cycle and with irreversible senescence induced by stress. They are not hydrolyzed by β-glucosidase, but are degraded by the CKX enzyme [9,10,17,18]. The primary products of CK biosynthesis (ppbCKs) are inactive or very low active [19,20].

Numerous studies [21–23] have highlighted the close correlation between nitrogen nutrition and CK content in plants and have shown that nitrates are the major factors regulating the gene expression of adenosine phosphate-isopentenyltransferase (IPT), a key enzyme in CK biosynthesis [24,25]. The translocation of xylem CKs increases in response to elevated nitrogen status [26]. The overall metabolic adaptation of a plant is affected by its N status and especially its C/N status [27]. With changing N content in the tissue, the above authors have observed changes in the metabolite composition of carbohydrates, organic acids and amino acids. The N assimilation can be disrupted by As [28]. The As toxicity caused the reduction in NO_3_^−^ and NO_2_^−^ content in potato plants [29] and reduction of NO_3_^−^ content in the roots, rhizomes and fronds of As-hyperaccumulator *Pteris vittata* and As-non-hyperaccumulator *P*. *ensiformis* [30]. The enzymatic activities of nitrate reductase, nitrite reductase and glutamate dehydrogenase was disturbed by As in rice [31], pea [32], *Pityrogramma calomelanos* [33] and potato [29]. Further, As toxicity caused the accumulation of NH_4_^+^ in plants [29,31]. Ammonium in high levels becomes toxic to plants since it can cause inhibition of the net assimilation of CO_2_, damage to the structure of chloroplasts, nutritional deficiencies, hormonal disequilibrium, etc. [34].

Our previous results show that N flow via amino acids can change dramatically in response to stress caused by toxic elements [35–37]. *Pityrogramma calomelanos* tolerates high concentrations of As due to its ability to upregulate the biosynthesis of amino acids and antioxidants, without greatly disturbing central carbon metabolism [33]. Oxidative stress activates senescence associated with remobilization of basic nutrients C, N, P and S [38]. in the drop of photosynthetic performance under As(V) toxicity is attributed to a decrease in maximal rate of RuBisCO carboxylation, which is reversed by nitrogen availability. Arsenic toxicity was found to be compensated primarily by upregulation of photosynthetic parameters and key nitrogen metabolizing enzymes [36]. In this experiment with *Solanum lycopersicum* L., major photosynthetic criteria like net photosynthetic rate and maximum quantum efficiency of photosystem II were significantly reduced under As(V) stress.

Cytokinins are responsible for increasing plants’ tolerance to various environmental stresses [1,39–42]. They are effective in delaying the breakdown of chlorophyll, suggesting that they may play a role in maintaining plants’ photosynthetic apparatus. It has also been reported that photosynthesis improves under nitrogen availability, as efficient N-metabolism helps to channel photosynthates into organic-nitrogen molecules, thereby preventing the feedback inhibition of photosynthesis [43]. A significant correlation was found between the frond endogenous CK *trans*-zeatin content in the As hyperaccumulator *Pteris vittata* and As, Pb, and Cd concentrations in the soil in a multi-metal pollution area [44]. The content of *tran*s-zeatin was also increased significantly in the fronds of As-hyperaccumulator *Pteris cretica* var. nervosa, but decreased in As-non-hyperaccumulator *Pteris ensiformis* in the presence of As(V) in the growth medium [45]. The content of many endogenous CKs in fronds of *P*. *ensiformis* was decreased by As and is likely to resist the phytotoxicity imposed by As [46]. Arsenate also caused severe depletion of endogenous CKs in the model plant *Arabidopsis thaliana* [47]. According to these results, changes of CK levels in response to As stress are thus a central factor in As tolerance and accumulation in plants.

In this study, we used an As-hyperaccumulator *P*. *cretica* var. Albo-lineata and Parkerii and the non-As-hyperaccumulator *Pteris straminea* to investigate effect of As contamination on N metabolism, CKs and photosynthesis. The aim of this study was to: (1) compare the content of As, total N, nitrate, CKs and amino acids (storage and transport of N) in the hyperaccumulator ferns to those of the non-hyperaccumulator; (2) investigate the effect of As on individual endogenous CK forms and amino acids; (3) obtain the information on the effect of As on net photosynthetic rate and chlorophyll fluorescence; and (4) reveal the relationship between individual endogenous CK forms and C and/or N metabolism.

## Materials and methods

The ferns, As-hyperaccumulating *P*. *cretica* L. var. Albo-lineata (*Pc*-A) and var. Parkerii (*Pc*-P) and As-non-hyperaccumulating *P*. *straminea* Mett. ex Baker (*Ps*), were cultivated in a pot experiment. Ferns at the 10-fronds stage were purchased from the garden centre Tulipa Praha in the Czech Republic. The experiment was carried out under greenhouse conditions (natural photoperiod; temperature 22─24°C; relative humidity ~60%) for 23 weeks. Five kg Haplic Chernozem from a non-polluted area in Prague-Suchdol, Czech Republic (total organic carbon 1.83%, cation-exchange capacity 258 mmol·kg^-1^, pH_KCl_ 7.1, total As 16 mg·kg^-1^, water soluble As 0.15 mg·kg^-1^ and As extraction efficiency 20%) was used per pot. Each kg soil was mixed with 0.5 g N, 0.16 g P and 0.4 g K (applied as NH_4_NO_3_ and K_2_HPO_4_). Then, the soil was spiked with 20 (As1) or 100 (As2) mg As·kg^-1^. The background soil As content was not included in applied As dose and the difference between control (without As application) and As1 or As2 treatment equalled the As spiked dose plus 20% from total As content—23.2 or 103.2 mg As·kg^-1^, respectively. Arsenic was added as a solution of Na_2_HAsO_4_ and was thoroughly mixed with the soil. The experiment was conducted in triplicate. Each pot contained one plant.

Following harvesting, the aboveground biomass was partitioned, with one portion being immediately frozen in liquid nitrogen and stored at –80°C until analysis for phytohormones and amino acids, while the other portion was oven-dried to constant weight (three days at 40°C) and homogenized for element analysis.

### Analysis of elements in plant biomass

#### Arsenic

Homogenized material (0.5±0.05 g of dry weight) was digested in 10 mL of a mixture of HNO_3_ and H_2_O_2_ (4:1, v/v) in an Ethos 1 device (MLS GmbH, Leutkirch im Allgäu, Germany). After cooling the digested sample was diluted to 50 mL with demineralized water. The content of As was determined using an Agilent 720 inductively coupled plasma-optical emission spectrometer (ICP-OES; Agilent Technologies Inc., Santa Clara, CA, USA). A certified reference material (CRM NIST 1573a Tomato leaves) was mineralised under the same conditions for quality assurance.

#### Total nitrogen (N_T_)

The plant material (1±0.05 g of dry weight) was decomposed by a liquid ashing procedure in H_2_SO_4_ solution (1:20 w/v) and analyzed by the Kjeldahl method using a Vapodest 50s distillation system (Gerhardt Gmbh & Co. KG., Königswinter, Germany) as previously described [24].

#### Nitrate nitrogen (N-NO_3_^-^)

Homogenized plant samples (0.5±0.05 g of dry weight) were extracted with 50 mL of deionized water at room temperature. The samples were shaken for 2 h (275 rpm; shaker GFL 3006) and filtered through filter paper for qualitative analysis (KA 2, 80 g·m^−2^, Perštejn, Czech Republic). The content of N-NO_3_^-^ was determined by segmented flow analysis using infrared detection on a SKALARplusSYSTEM (Skalar, the Netherlands).

### Analysis of cytokinins

Phytohormones were extracted, purified and quantified according to previously published methods [8]. In summary, the samples were homogenized with a MM301 ball mill (Retsch, Prague, Czech Republic) and stable isotope-labelled internal standards. Extraction occurred in cold (−20°C) methanol/water/formic acid (15:4:1, v/v/v). The extracts were purified using an Oasis-MCX mixed-mode solid-phase extraction (SPE) column (Waters Corp., Milford, MA, USA). The resulting two fractions contained acidic and basic phytohormones. The fraction of basic phytohormones, including CKs, was analyzed using a Dionex UltiMate 3000 high-performance liquid chromatography (HPLC) system (Thermo Fisher Scientific, Waltham, MA, USA,) coupled to a 3200 Q TRAP hybrid triple quadrupole/linear ion trap mass spectrometer (Applied Biosystems, Waltham, MA, USA) in multiple reaction monitoring (MRM) mode. Data processing and hormone quantification were done using Analyst 1.5 software (Applied Biosystems).

### Analysis of amino acids

Free amino acids (AAs) were extracted, derivatized and quantitated according to previously published method [37], with minor modifications. In brief, each sample (1g of fresh weight) was extracted with 15 mL of methanol and H_2_O (7:3, v/v). The extracts were derivatized using an EZ:faast kit (Phenomenex, Torrance, CA, USA). The prepared samples were analyzed on a Hewlett Packard 6890N/5975 MSD gas chromatography-mass spectrometry (GC-MS) system (Agilent technologies).

### Determination of chlorophyll fluorescence (Fv/Fm)

Chlorophyll fluorescence (μmol·m^-2^·s^-1^) and variable fluorescence (Fv)/maximal fluorescence (Fm) were measured using a modulated chlorophyll fluorometer OS1-FL (Opti-Sciences Inc., ADC BioScientific Ltd., Hoddesdon, UK). A fresh leaf was obscured by clipping for 20 minutes to establish a dark-adapted state. Chlorophyll fluorescence was realized using a 660 nm solid-state light source, with filters blocking radiation longer than 690 nm. Saturation of the photosystem being measured was achieved using a filtered 35 W halogen lamp (350─690 nm) with a 15,000 μmol·m^-2^·s^-1^ pulse for 0.8 seconds.

### Determination of net photosynthetic rate (P_N_)

The portable gas exchange system LCpro+ (ADC BioScientific, Ltd., Hoddesdon, UK) was used for *in situ* determination of the net photosynthetic rate (P_N_; μmol CO_2_·m^-2^·s^-1^). The measurements were conducted between 8:00 and 11:30 Central European Time. The duration of each individual measurement was 10 min after the establishment of steady-state conditions inside the measurement chamber. The conditions in the chamber were: 25°C, ambient CO_2_ concentration 550±50 μL·L^-1^, air-flow rate 205±30 μmol·s^-1^ and irradiance 650±50 μmol·m^-2^·s^-1^ of photosynthetically active radiation [48].

### Statistical analysis

All data were checked for homogeneity of variance and normality (Levene and Shapiro-Wilk tests). The collected data were evaluated with the non-parametric Kruskal-Wallis test, using Statistica 12.0 software (www.statsoft.com). A principal component analysis (PCA) using CANOCO 4.5 software [49] was applied to all collected data as a single set. Standardization of species was used because data of different characters were being analyzed together. PCA was used to correlate the analyzed data, similarity of species and sampling period visible from the complex data set. The results were visualized as a bi-plot ordination diagram using CanoDraw. Correlations were quantified using Pearson linear correlation (r; p < 0.05) with Statistica 12.0 software.

## Results

### Accumulation of As and its effect on frond biomass

**Fig 1:**
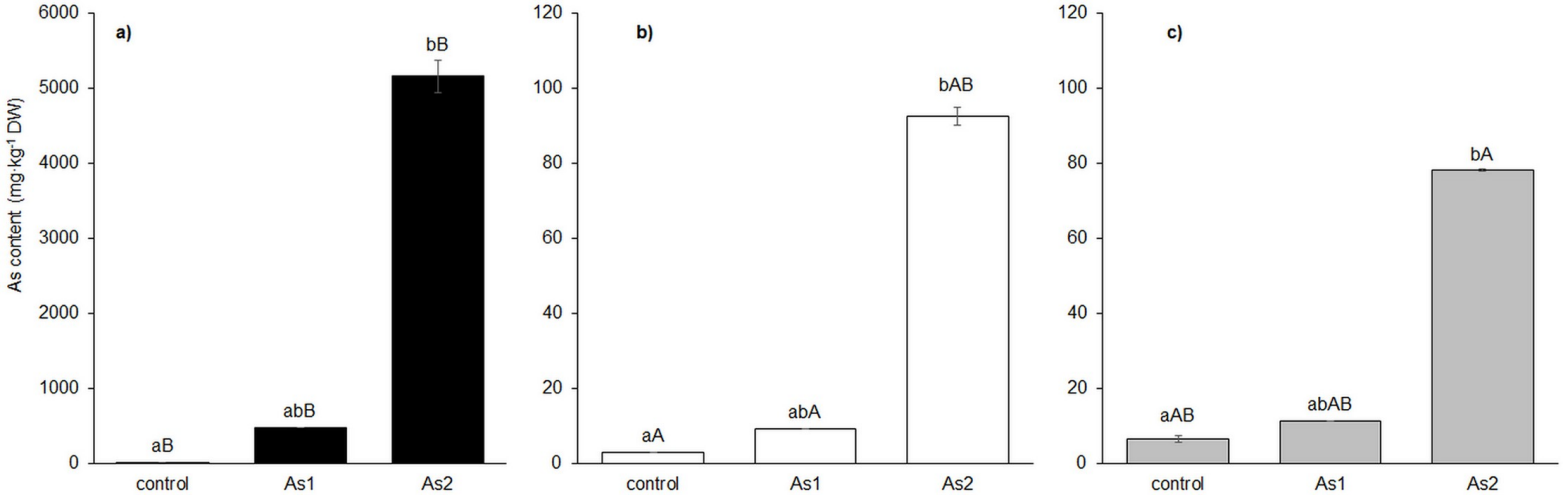
Accumulation of As in fronds of the As-hyperaccumulator ferns *P*. *cretica* var. Albo-lineata (a) and *P*. *cretica* var. Parkerii (b) and the As-non-hyperaccumulator fern *P*. *straminea* (c). Values are mean ± standard error (SE). Data with the same letter are not significantly different. Different letters indicate significant differences (p < 0.05) among variants of each fern (lower-case letters) and among the individual ferns of each variant (upper-case letters) according to the Kruskal-Wallis test. Treatment abbreviations: control– 0 mg As·kg^-1^ soil; As1–20 mg As·kg^-1^ soil; As2–100 mg As·kg^-1^ soil. The background soil As content is 16 mg As·kg^-1^ soil. The difference between control and individual As treatments is the spiked As dose plus the 20% As extraction efficiency.

The As concentration in the plant tissues of all of the ferns studied grew with increasing content in the soil (Fig 1). The highest As content was found in *Pc*-A and the results confirmed *Pc*-A as an As-hyperaccumulator. By contrast, the other As hyperaccumulating fern—*Pc*-P, did not show an ability to hyperaccumulate As in this experiment. Yet, our experiment with As content in the soil higher than that used in this experiment confirmed *Pc*-P as an As-hyperaccumulator (data in S1 Fig). As accumulation similar to that in *Pc*-P was found in *Ps* (Fig 1).

**Fig 2:**
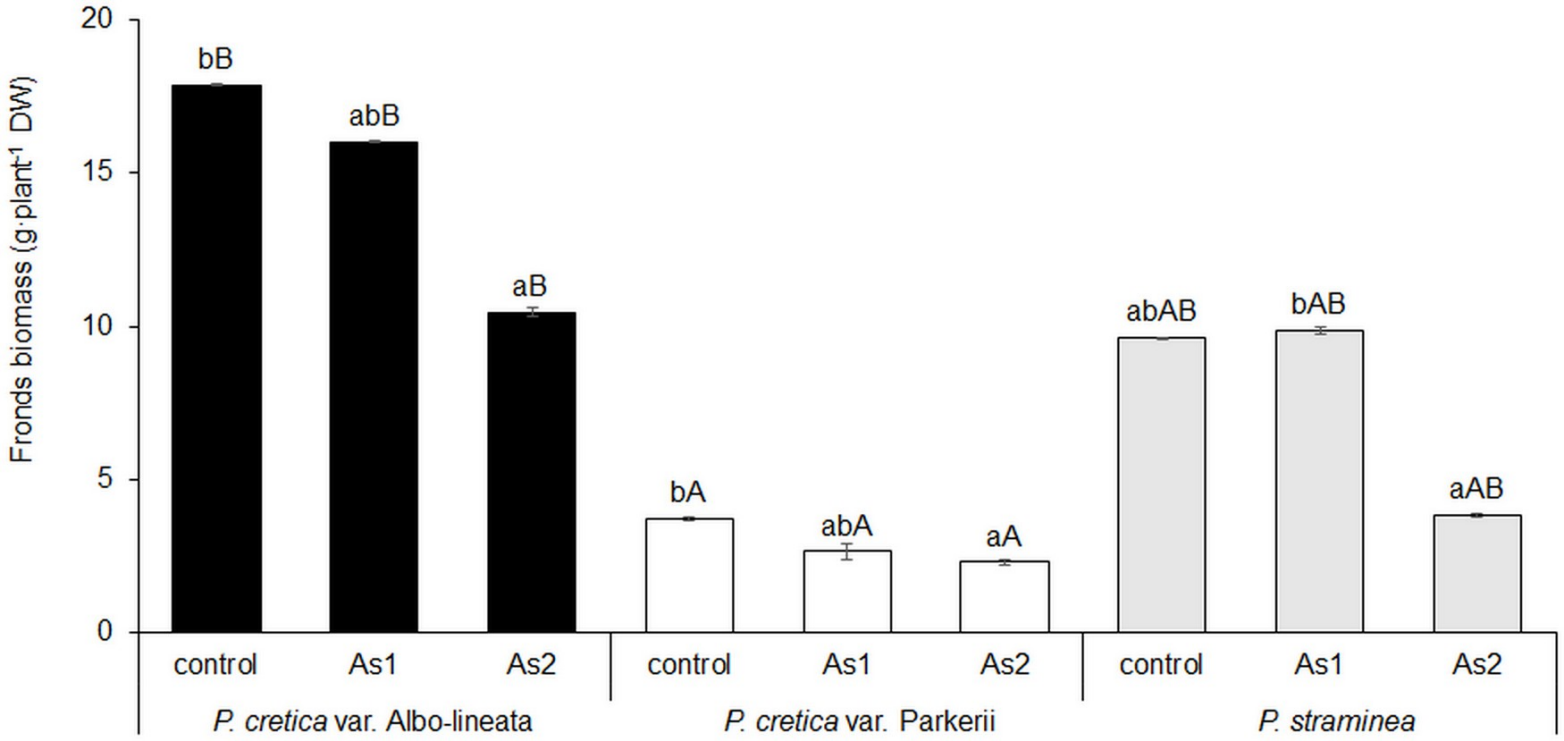
Changes in frond biomass of the As-hyperaccumulator ferns *P*. *cretica* var. Albo-lineata and *P*. *cretica* var. Parkerii and the As-non-hyperaccumulator fern *P*. *straminea* after 23 weeks of exposure to As. Values are mean ± standard error (SE). Data with the same letter are not significantly different. Different letters indicate significant differences (p < 0.05) among variants of each fern (lower-case letters) and among the individual ferns of each variant (upper-case letters) according to the Kruskal-Wallis test. Treatment abbreviations: control– 0 mg As·kg^-1^ soil; As1–20 mg As·kg^-1^ soil; As2–100 mg As·kg^-1^ soil. The background soil As content is 16 mg As·kg^-1^ soil. The difference between control and individual As treatments is the spiked As dose plus the 20% As extraction efficiency.

At harvest, the dry weight of the frond biomass of all tested ferns declined with increasing As supply in the soil (Fig 2); this decrease was significant in the case of the As2 treatments. The highest biomass yield was obtained from all treatments of *Pc*-A, while the lowest biomass yield was obtained from all treatments of *Pc*-P (Fig 2). A significant correlation between As content and biomass yield was calculated for all studied ferns (data in S1 Table).

### Effect of As on the regulation of the various CKs according to their structure and physiological roles

For the purpose of this study, the CKs were placed into five functionally different groups according to their structure and physiological roles [19] (Table 1): (1) bioactive forms (bCKs; CK free bases); (2) inactive or weakly active forms (dCKs; CK *N*-glucosides); (3) transport forms (tCKs; CK ribosides); (4) storage forms (sCKs; *O-*glucosides); and (5) primary products of CK biosynthesis (ppbCKs; CK nucleotides).

**Table 1:** Content of cytokinins (pmol·g^-1^ fresh weight) in fronds of the As-hyperaccumulator ferns *P*. *cretica* var. Albo-lineata (*Pc*-A) and *P*. *cretica* var. Parkerii (*Pc*-P) and the As-non-hyperaccumulator fern *P*. *straminea* (*Ps*) after 23 weeks of exposure to As.

	Variants					
	Control		As1		As2	
	$\bar{x}$ ± SE	%*	$\bar{x}$ ± SE	%*	$\bar{x}$ ± SE	%*
*Pc*-A						
ΣCKs	126.6±2.5^aAB^	-	176.7±0.6^abAB^	-	211.5±5.8^bA^	-
bCKs	2.5±0.3^aA^	2.0	2.5±0.2^aA^	1.4	10.7±1^aA^	5.1
tCKs	3.8 ± 0.01^aA^	3.0	6.2±0.1^abA^	3.5	15.6±0.6^bA^	7.4
dCKs	9.3±0.3^aAB^	7.4	11.7±0.01^bAB^	6.6	7.2±0.4^abA^	3.4
sCKs	107.2±2.6^aAB^	84.7	154.9±1.4^abAB^	87.7	171.1±6.5^bAB^	80.9
ppbCKs	3.7±0.6^abA^	2.9	1.5±0.4^aA^	0.8	6.9±0.1^bAB^	3.3
***Pc*-P**						
ΣCKs	383.2±41.8^abB^	-	800.4±20.2^bB^	-	378.0±49.8^aA^	-
bCKs	3.0±0.7^aA^	0.8	4.7±0.7^abA^	0.6	72.2±7.7^bB^	19.1
tCKs	12.4±0.9^aB^	3.2	11.3±0.5^aAB^	1.4	15.0±2.9^aA^	4.0
dCKs	80.9±24.2^bB^	21.1	35.8±2.1^abB^	4.5	35.9±4.4^aA^	9.5
sCKs	271.2±15.4^abB^	70.8	734.1±20.6^bB^	91.7	252.1±34.7^aB^	66.7
ppbCKs	15.6±0.7^bB^	4.1	14.5±0.6^abB^	1.8	2.7±0.003^aA^	0.7
***Ps***						
ΣCKs	29.7±3.1^aA^	-	42.8±3^abA^	-	209.9±10^bA^	-
bCKs	1.9±0.06^aA^	6.3	5.2±0.2^abA^	12.3	21.9±0.2^bAB^	10.4
tCKs	7.0±0.6^aAB^	23.4	15.5±1.1^abB^	36.3	45.3±0.9^bA^	21.6
dCKs	1.6±0.1^abA^	5.3	1.0±0.03^aA^	2.2	7.1±0.3^bA^	3.4
sCKs	14.1±4^aA^	47.3	12.8±1.7^aA^	30.0	124.5±10.8^aA^	59.3
ppbCKs	5.3±0.02^aAB^	17.7	8.2±0.4^abAB^	19.3	11.2±0.2^bB^	5.4

The values are mean ± standard error (SE). Data with the same letter were not significantly different. Different letters indicate significant differences (p < 0.05) among variants of each fern (lower-case letters) and among the individual ferns of each variant (upper-case letters) according to the Kruskal-Wallis test. Treatment abbreviations: control– 0 mg As·kg^-1^ soil; As1–20 mg As·kg^-1^ soil; As2–100 mg As·kg^-1^ soil. The background soil As content– 16 mg As·kg^-1^ soil. The difference between control and individual As treatments equals the spiked As dose plus the 20% As extraction efficiency. ΣCKs–total cytokinins; bCKs–bioactive cytokinin forms; dCKs–inactive (or weakly active) cytokinin forms; tCKs–transport cytokinin forms; sCKs–storage cytokinin forms; ppbCKs, primary products of cytokinin biosynthesis.

*content of individual cytokinin groups as percentage of all cytokinins.

The total sum of all cytokinins (ΣCKs) increased in—As1-treated *Pc*-P ferns (Table 1). By contrast, in As2 treatments an increase of ΣCKs was found for *Pc*-A and *Ps*. Whereas *Ps* showed a dramatic increase in ΣCK content compared to the control treatment, the increase for *Pc*-A was rather low. The total sum of all CKs in *Pc*-P was significantly higher in comparison with *Pc*-A and *Ps* in control and As1 treatments.

The highest representation in the total CK pool for *Pc*-A and *Pc*-P was that of sCKs, with 80.9–87.7% and 66.7–91.7%, respectively. By contrast, they only represented 30.0–59.3% of ΣCKs in *Ps*. In *Ps*, tCKs were highly abundant, representing the second most plentiful CK group (Table 1). On the other hand, the content of tCKs in *Pc*-A and *Pc*-P was very low. The ppbCKs represented a significant CK group in *Ps*, especially in control and As1-treated plants. However, the portion of ppbCKs in the total CK pool was considerably lower in plants treated with As2 (Table 1).

**Fig 3:**
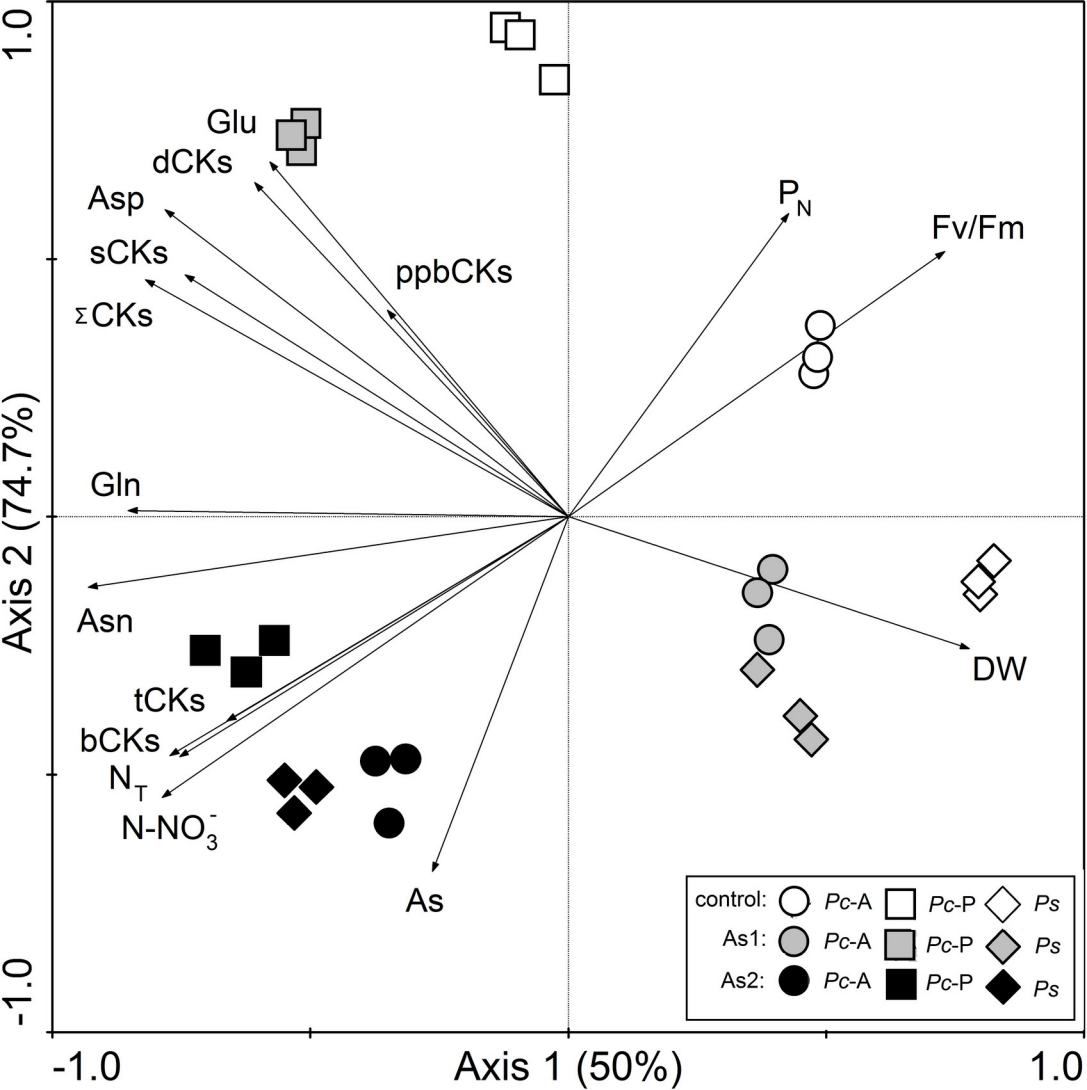
Ordination bi-plot of selected parameters in fronds of the As-hyperaccumulator ferns *P*. *cretica* var. Albo-lineata (*Pc*-A) and *P*. *cretica* var. Parkerii (*Pc*-P) and the As-non-hyperaccumulator fern *P*. *straminea* (*Ps*). The first axis of the PCA explains 50%, the first two axes 74.7% and the first four axes together 95% of the variability of all analyzed data. The length and the direction of the vectors indicate the strength of the vector effect and the correlation between the vectors, respectively. A long vector for a particular variable indicates that it is greatly affected the results of the analysis, while the opposite was true for a short vector. An angle of <90° between the vectors indicates that they are positively correlated. The angle of >90° between two vectors indicates that they are not positively correlated. The effect of the individual treatment differs between ferns. In *Pc*-P, in contrast to *Pc*-A and *Ps* ferns, the data for As1 treatment are clearly separated from all others. This indicates a large effect of As1 treatment on all of the recorded data in *Pc*-P and a minimal effect for *Pc*-A and *Ps*. The data for control and As2 treatments are located in different parts of the diagram, indicating a substantial effect of As2 application. Treatment abbreviations: control– 0 mg As·kg^-1^ soil; As1–20 mg As·kg^-1^ soil; As2–100 mg As·kg^-1^ soil. The background soil As content is 16 mg As·kg^-1^ soil. The difference between control and individual As treatments is the spiked As dose plus the 20% As extraction efficiency. Parameter abbreviations: DW–yield of dry frond biomass; As–arsenic; P_N_−net photosynthetic rate; Asp–aspartic acid; Asn–asparagine; Glu–glutamic acid; Gln–glutamine; N_T_−total nitrogen; N-NO_3_^-^ –nitrate nitrogen; Fv/Fm–chlorophyll fluorescence; ΣCKs–total cytokinins; bCKs–bioactive cytokinin forms; dCKs–inactive (or weakly active) cytokinin forms; tCKs–transport cytokinin forms; sCKs–storage cytokinin forms; ppbCKs–primary products of cytokinin biosynthesis.

Increased As soil contamination resulted in an enhanced accumulation of bCKs and tCKs in plants (Table 1). A significant effect of As2 treatment on tCKs was shown in *Pc*-A and *Ps*. The percentage of bCKs varied in all ferns, because CK homeostasis was regulated by As accumulation in fronds, as indicated by the results of PCA and the correlation (Fig 3, S1 Table). Therefore, increased As accumulation enhanced bCK content in all ferns (Table 1, S1 Table) and a significant effect of As2 treatment was shown in *Pc*-P and *Ps*. Differences among ferns in terms of tCKs were shown in control and As1 treatment, while in terms of bCKs they were perceptible only in As2 treatment (Table 1). Both bCKs and tCKs have generally been found to improve plant adaptation and promote plant growth [1,9–11,19]. This phenomenon was not shown for the ferns under study. Our results indicated a reduction in frond biomass (Fig 2) and no increased C assimilation. A negative correlation between bCKs and dry biomass yield was confirmed in all ferns; however, the relationship between tCKs and dry biomass yield was confirmed only for *Pc*-A and *Ps* (data in S2 Table).

Arsenic toxicity also instigated a different dCKs variation in all ferns. The difference between the ferns in terms of dCK content was not found in variants with the highest As contamination level (Table 1). The effect of As treatments on dCKs and its trend was ambiguous. Differences among ferns in terms of sCKs and ppbCKs were shown in all treatments (Table 1). The content of sCKs was affect by As2 treatment in *Pc*-A and by As1 treatment in *Pc*-P. Increase of ppbCks by As2 treatment was shown in *Pc*-A and *Ps*, while a decrease appeared in *Pc*-P. The differences in the levels of the individual CK groups were consistent with the distinction between As-hyperaccumulator and As-non-hyperaccumulator ferns.

### Effect of As on assimilation of C and N

**Fig 4:**
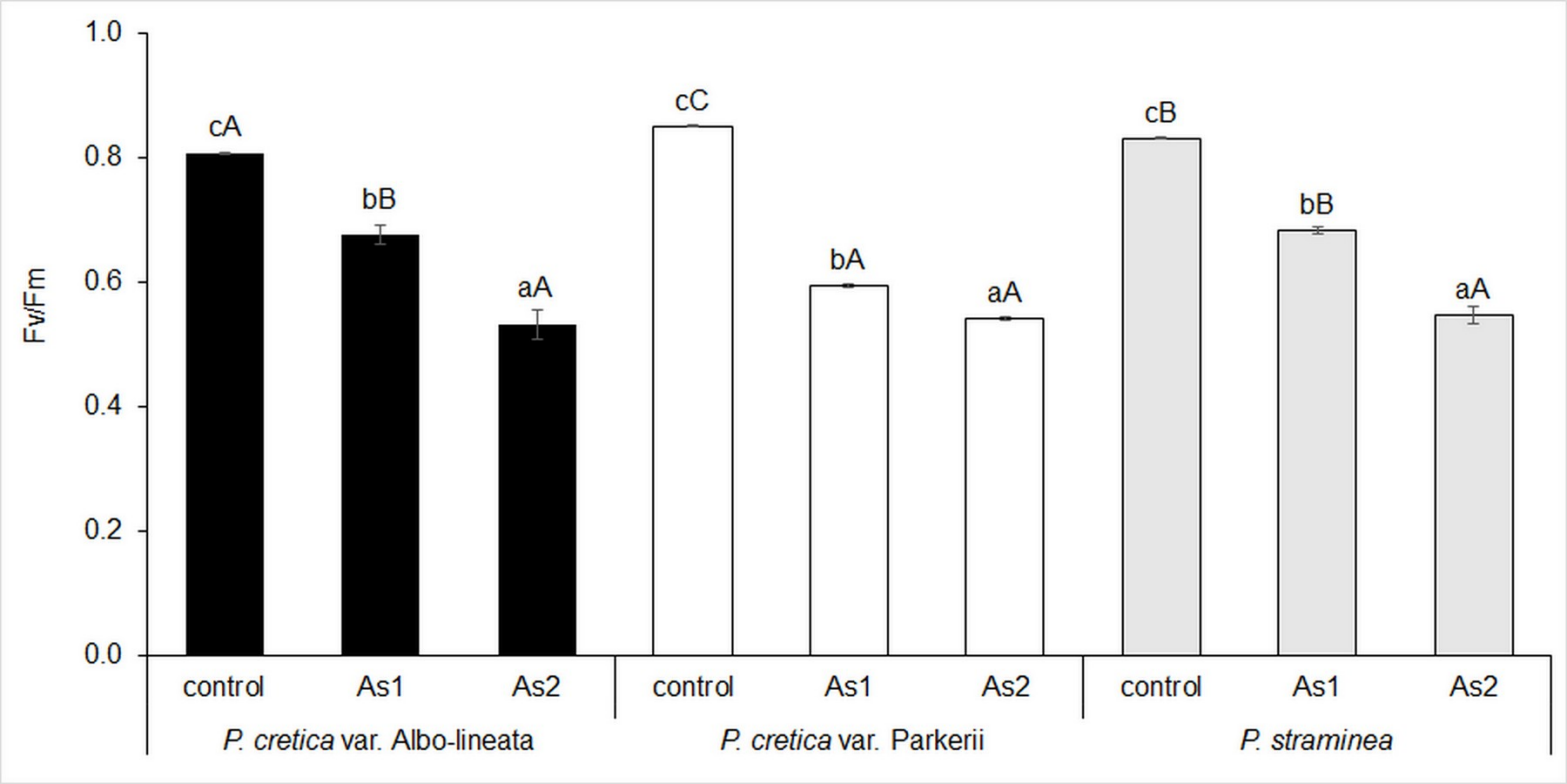
Changes in chlorophyll fluorescence (Fv/Fm) in fronds of the As-hyperaccumulator ferns *P*. *cretica* var. Albo-lineata and *P*. *cretica* var. Parkerii and the As-non-hyperaccumulator fern *P*. *straminea* after 23 weeks of exposure to As. Values are mean ± standard error (SE). Data with the same letter are not significantly different. Different letters indicate significant differences (p < 0.05) among variants of each fern (lower-case letters) and among the individual ferns of each variant (upper-case letters) according to the Kruskal-Wallis test. Treatment abbreviations: control– 0 mg As·kg^-1^ soil; As1–20 mg As·kg^-1^ soil; As2–100 mg As·kg^-1^ soil. The background soil As content is 16 mg As·kg^-1^ soil. The difference between control and individual As treatments is the spiked As dose plus the 20% As extraction efficiency.

**Fig 5:**
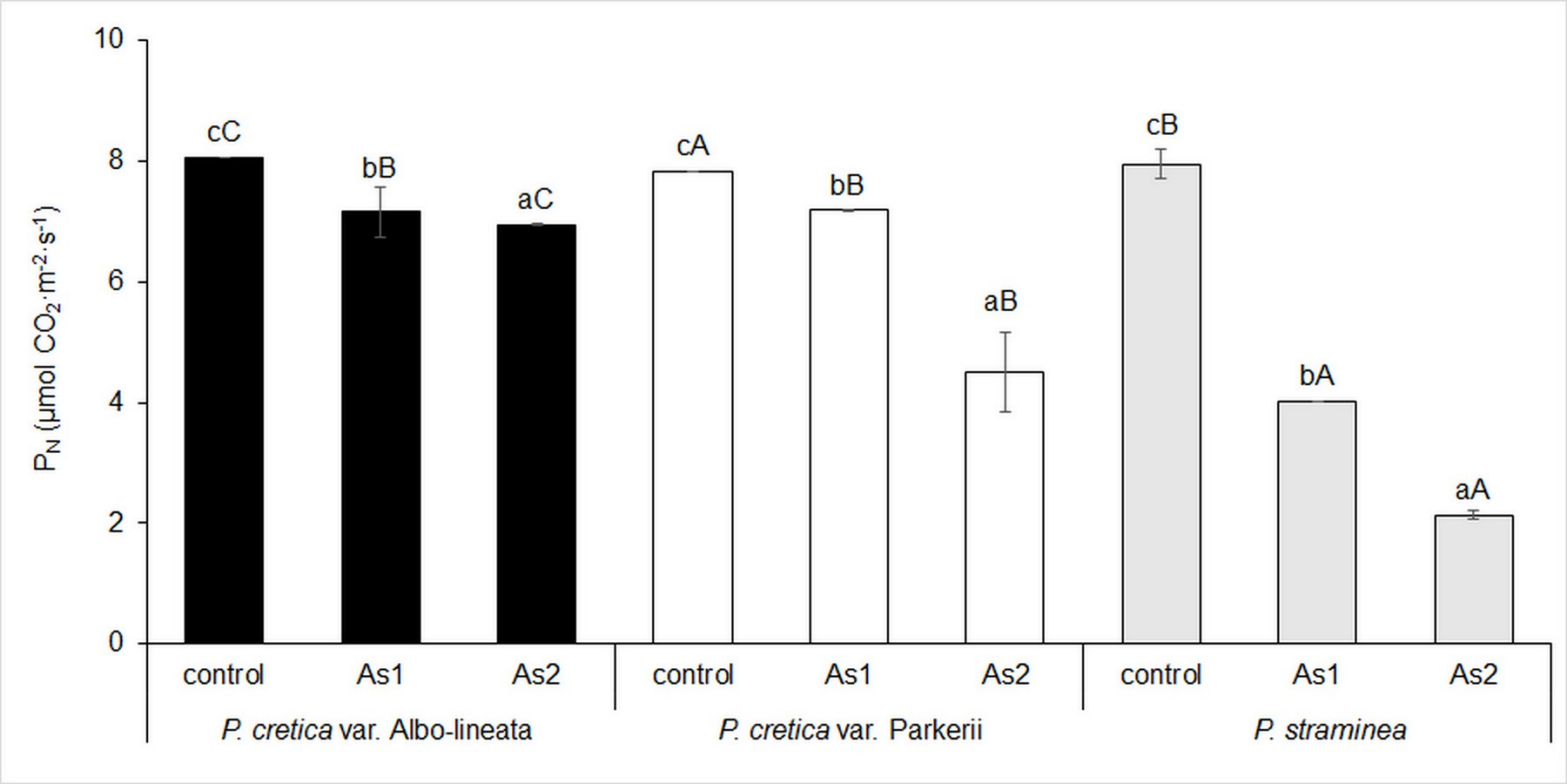
Changes in net photosynthetic rate (P_N_) in fronds of the As-hyperaccumulator ferns *P*. *cretica* var. Albo-lineata and *P*. *cretica* var. Parkerii and the As-non-hyperaccumulator fern *P*. *straminea* after 23 weeks of exposure to As. Values are mean ± standard error (SE). Data with the same letter are not significantly different. Different letters indicate significant differences (p < 0.05) among variants of each fern (lower-case letters) and among the individual ferns of each variant (upper-case letters) according to the Kruskal-Wallis test. Treatment abbreviations: control– 0 mg As·kg^-1^ soil; As1–20 mg As·kg^-1^ soil; As2–100 mg As·kg^-1^ soil. The background soil As content is 16 mg As·kg^-1^ soil. The difference between control and individual As treatments is the spiked As dose plus the 20% As extraction efficiency.

The assimilation of C in ferns was assessed on the basis of chlorophyll fluorescence (Fv/Fm) and net photosynthetic rate (P_N_). For all ferns, the selected parameters were inhibited with increasing As content (Figs 4 and 5). Significant differences were also noted among the control treatments of the cultivated ferns. The most significant differences between variants and ferns were detected in the P_N_ value. There was a significant negative correlation between As levels and P_N_ in all plants, but a negative correlation between As and Fv/Fm was found only for *Pc*-A and *Ps* (data in S1 Table). A close linear correlation was found in *Pc*-P, although it was not statistically significant (data in S1 Table).

**Fig 6:**
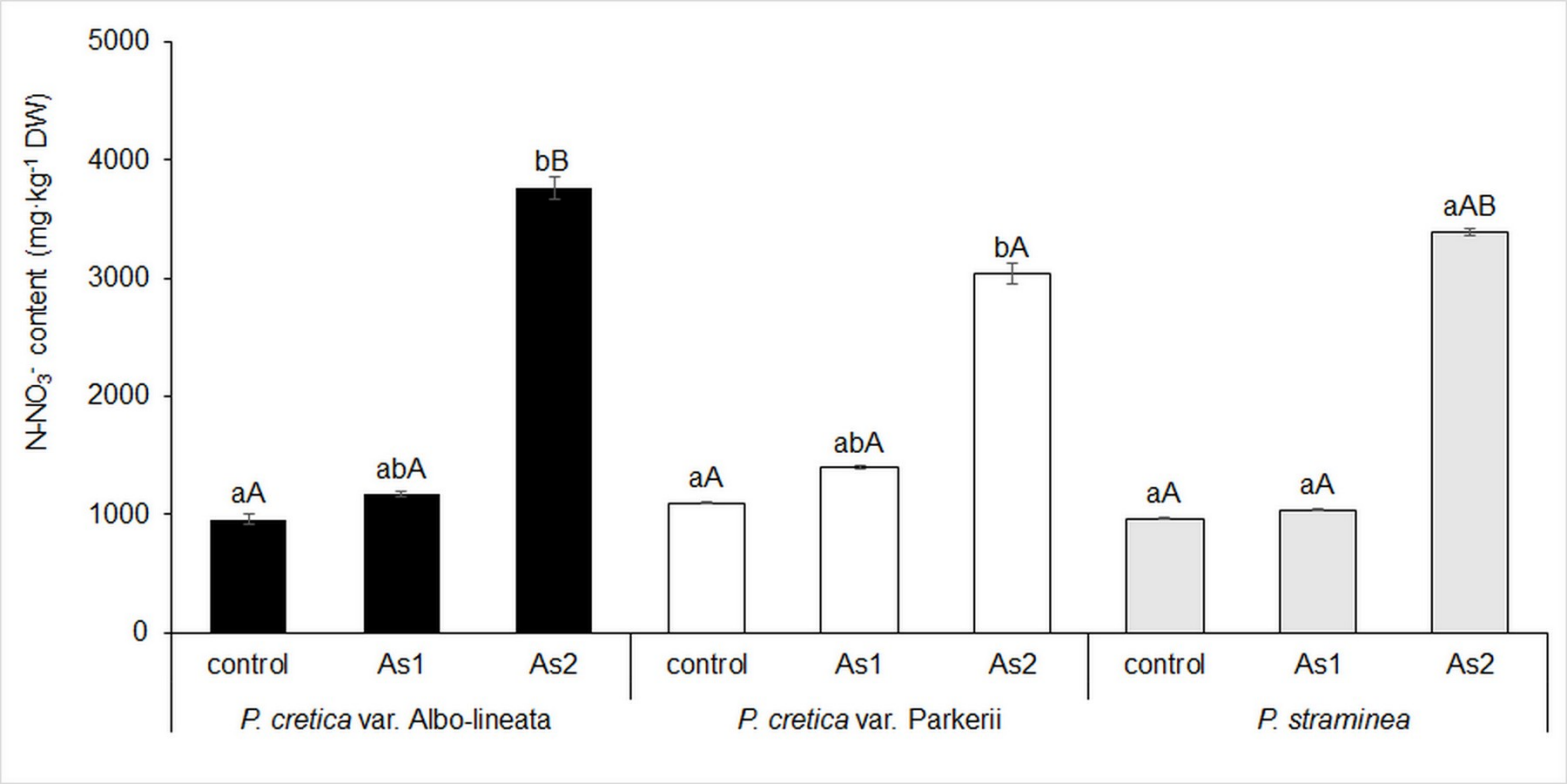
Changes in nitrate nitrogen (N-NO3-) content in fronds of the As-hyperaccumulator ferns *P*. *cretica* var. Albo-lineata and *P*. *cretica* var. Parkerii and the As-non-hyperaccumulator fern *P*. *straminea* after 23 weeks of exposure to As. Values are mean ± standard error (SE). Data with the same letter are not significantly different. Different letters indicate significant differences (p < 0.05) among variants of each fern (lower-case letters) and among the individual ferns of each variant (upper-case letters) according to the Kruskal-Wallis test. Treatment abbreviations: control– 0 mg As·kg^-1^ soil; As1–20 mg As·kg^-1^ soil; As2–100 mg As·kg^-1^ soil. The background soil As content is 16 mg As·kg^-1^ soil. The difference between control and individual As treatments is the spiked As dose plus the 20% As extraction efficiency.

The effect of As on N assimilation in ferns was assessed on the basis of N-NO_3_^-^ content, total N content and the content of transport amino acids and their amides as storage of amino group in plants. The N-NO_3_^-^ content of ferns did not significantly differ between control and As1 treatments, whereas it increased significantly (> three-fold) for *Pc*-A and *Pc*-P treated with As2 (Fig 6). Differences among ferns appeared in As2 treatment. The correlation between As and N-NO_3_^-^ was significant for all studied ferns (data in S1 Table).

**Fig 7:**
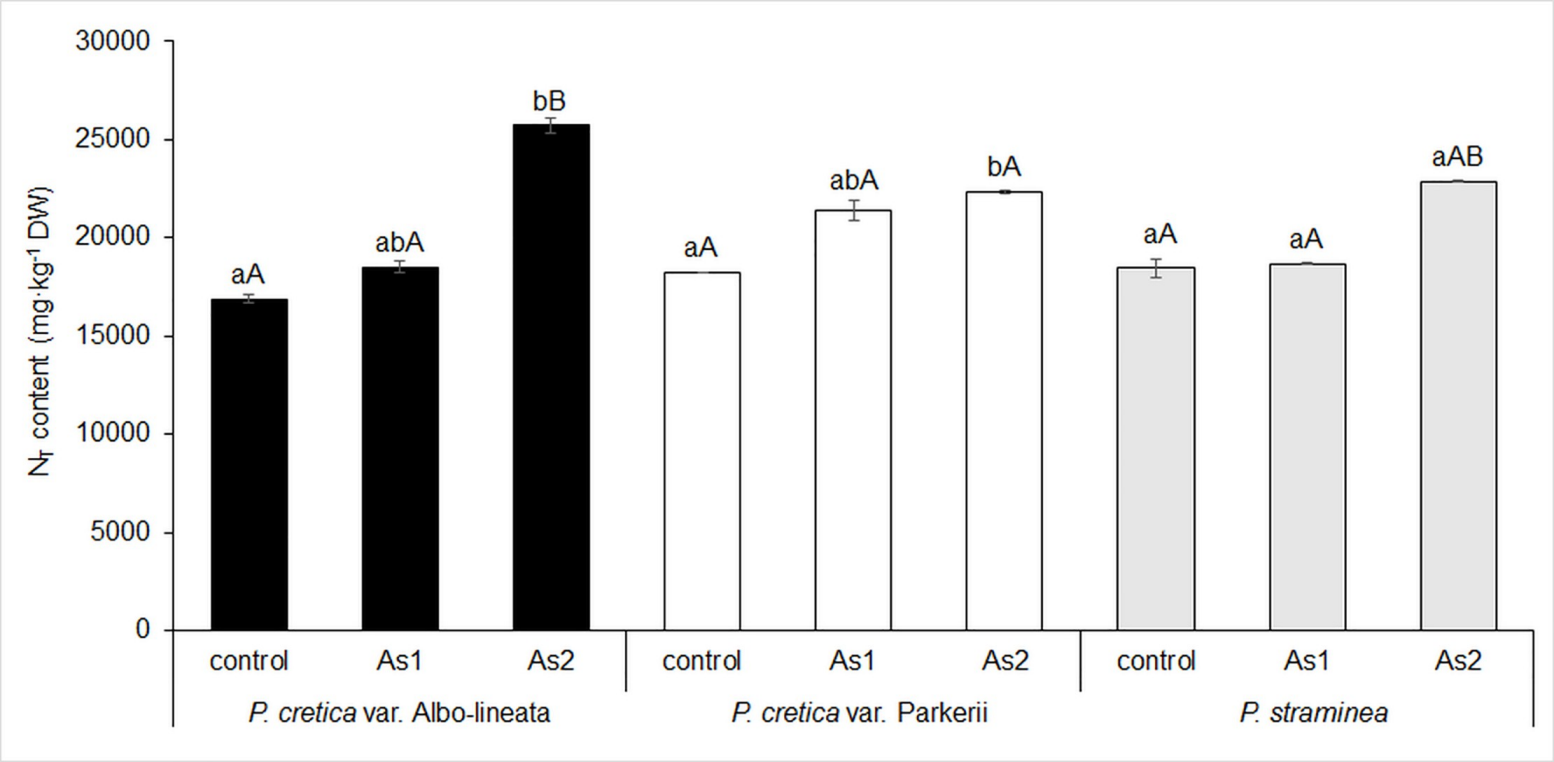
Changes in total nitrogen (N_T_) content in fronds of the As-hyperaccumulator ferns *P*. *cretica* var. Albo-lineata and *P*. *cretica* var. Parkerii and the As-non-hyperaccumulator fern *P*. *straminea* after 23 weeks of exposure to As. Values are mean ± standard error (SE). Data with the same letter are not significantly different. Different letters indicate significant differences (p < 0.05) among variants of each fern (lower-case letters) and among the individual ferns of each variant (upper-case letters) according to the Kruskal-Wallis test. Treatment abbreviations: control– 0 mg As·kg^-1^ soil; As1–20 mg As·kg^-1^ soil; As2–100 mg As·kg^-1^ soil. The background soil As content is 16 mg As·kg^-1^ soil. The difference between control and individual As treatments is the spiked As dose plus the 20% As extraction efficiency.

N uptake by ferns was affected by As contents, as shown using linear correlation (data in S1 Table). Total nitrogen (N_T_) content grew in all ferns with increasing As content in the soil (Fig 7). The differences between control and As1 treatments of all tested plants were not significant. However, significant differences were revealed between control and As2-treated plants for *Pc*-A and *Pc*-P, with 52% and 23% increases in N_T_, respectively. Differences in N_T_ among ferns were found in As2 treatment. The correlation between N_T_ and N-NO_3_^-^ contents in all ferns was significant (r = 0.76─0.99; p = 0.000–0.017), indicating an association with nitrogen metabolism. The contents of N-NO_3_^-^ and N_T_ were correlated with reduced dry frond biomass in all ferns (r = -0.76–0.99, p = 0.000–0.017 and r = -0.99, p = 0.000, respectively).

**Fig 8:**
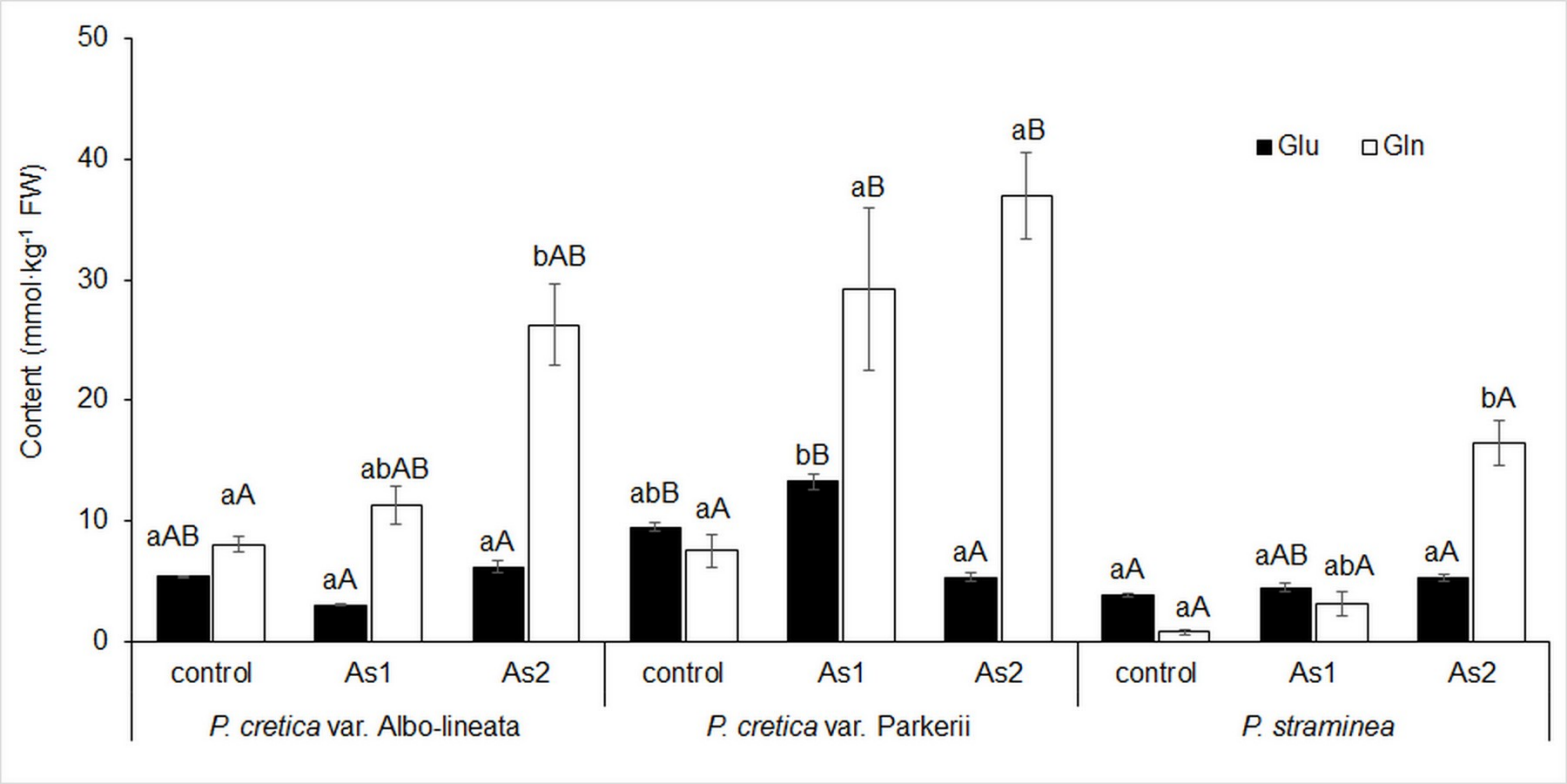
Changes in glutamic acid (Glu) and glutamine (Gln) content in fronds of the As-hyperaccumulator ferns *P*. *cretica* var. Albo-lineata and *P*. *cretica* var. Parkerii and the As-non-hyperaccumulator fern *P*. *straminea* after 23 weeks of exposure to As. Values are mean ± standard error (SE). Data with the same letter are not significantly different. Different letters indicate significant differences (p < 0.05) among variants of each fern (lower-case letters) and among the individual ferns of each variant (upper-case letters) according to the Kruskal-Wallis test. Treatment abbreviations: control– 0 mg As·kg^-1^ soil; As1–20 mg As·kg^-1^ soil; As2–100 mg As·kg^-1^ soil. The background soil As content is 16 mg As·kg^-1^ soil. The difference between control and individual As treatments is the spiked As dose plus the 20% As extraction efficiency.

The changes in N metabolism were also reflected in the levels of transport amino acids (tAAs), glutamic acid (Glu) and aspartic acid (Asp) as well as their storage amides (sAAs), glutamine (Gln) and asparagine (Asn). Gln and Asp represented the key amino acids in the biosynthesis of CKs, Glu was a key amino acid for chlorophyll synthesis. The close correlation between N_T_ content and Glu and Asp was only shown in *Ps* (r = 0.77, p = 0.015 and r = 0.98, p = 0.000, respectively). For all ferns, correlations were seen between the contents of N_T_ and sAAs (r = 0.76–0.97, p = 0.000–0.017) as well as between those of N-NO_3_^-^ and sAAs (r = 0.70–0.99, p = 0.000–0.035). The changes in Glu in ferns were ambiguous (Fig 8). Differences among ferns in Glu were revealed in control and As1 treatments. However, the correlation between As and Glu was only confirmed for *Pc*-P and *Ps* (data in S1 Table). Gln contents in the As treatments of both *Pc-*A and *Pc-*P plants were higher than those of Glu, while for the *Ps* plants, Gln levels were only higher in the As2 treatment. A significant effect of As2 treatment on Gln was shown in *Pc*-A and *Ps* (Fig 8). Both As treatments showed differences among ferns. The lowest contents of Glu and Gln were found in *Ps* plants. As treatments of *Pc*-A also resulted in an absolute increase in Gln levels, while the Gln content in *Ps* was increased only in the As2 treatment (Fig 8).

An increase in Gln content depending on As level was demonstrated for *Pc*-A and *Ps* (data in S1 Table). The Glu/Gln ratio, an indicator of primary N assimilation, diminished with increasing content of As in plants (Table 2). The highest Glu/Gln ratios were found in As-non-hyperaccumulator *Ps* in contrast to the As-hyperaccumulators *Pc*-A and *Pc*-P.

**Table 2:** Glu/Gln ratio in fronds of the As-hyperaccumulator ferns *P*. *cretica* var. Albo-lineata (*Pc*-A) and *P*. *cretica* var. Parkerii (*Pc*-P) and the As-non-hyperaccumulator fern *P*. *straminea* (*Ps*) after 23 weeks of exposure to As.

		Ferns		
Variants		*Pc*-A	*Pc*-P	*Ps*
Control	$\bar{x}$ ± SE	0.679±0.068	1.340±0.237	5.580±1.620
	%*	100	100	100
As1	$\bar{x}$ ± SE	0.278±0.031	0.501±0.104	1.60±0.291
	%*	41.0	37.4	28.6
As2	$\bar{x}$ ± SE	0.243±0.032	0.148±0.023	0.331±0.031
	%*	35.9	11.1	5.9

Values are mean ± standard error (SE).

* Glu/Gln ratio, where control corresponds to 100%.

**Fig 9:**
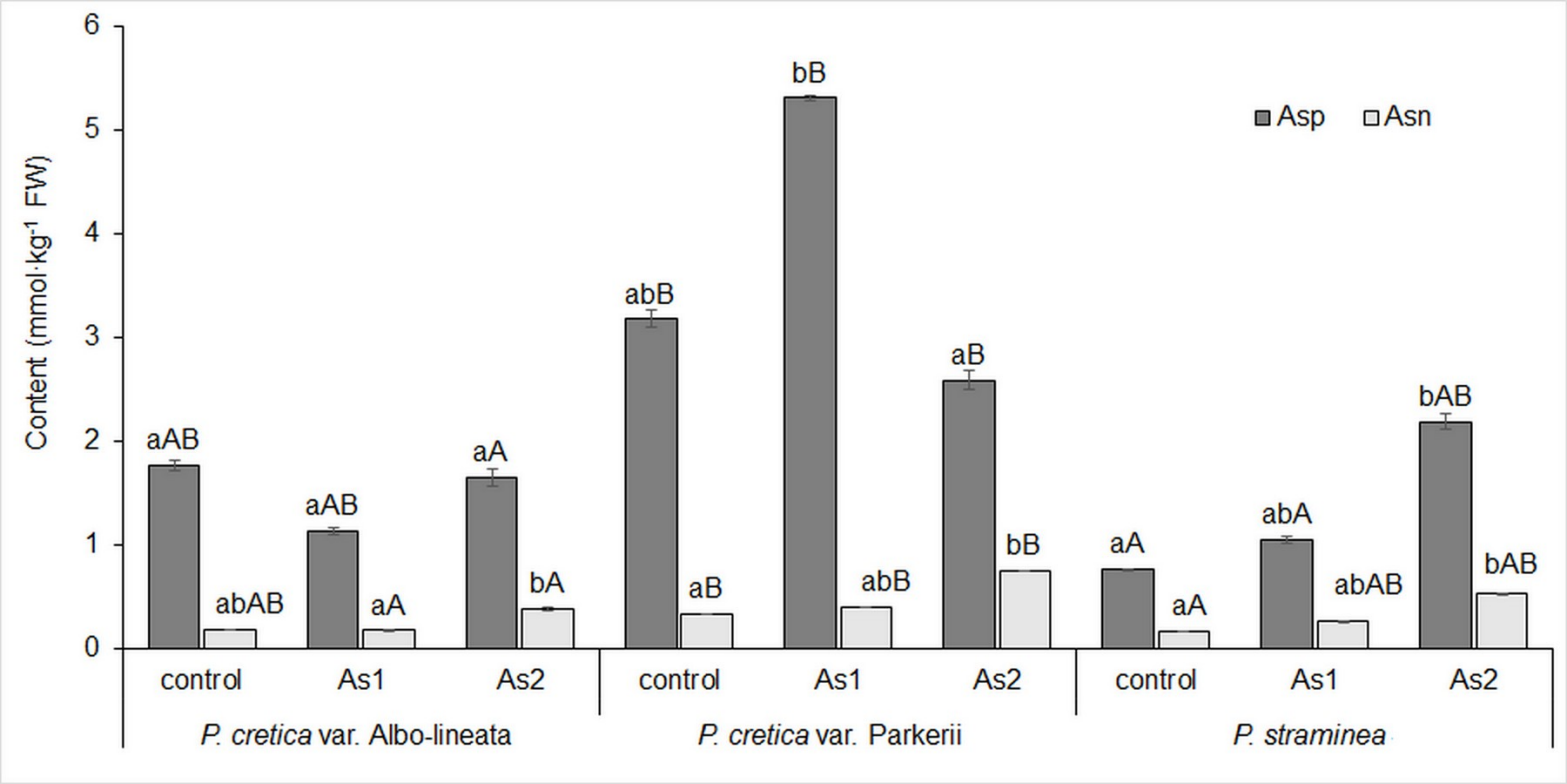
Changes in aspartic acid (Asp) and asparagine (Asn) content in fronds of the As-hyperaccumulator ferns *P*. *cretica* var. Albo-lineata and *P*. *cretica* var. Parkerii and the As-non-hyperaccumulator fern *P*. *straminea* after 23 weeks of exposure to As. Values are mean ± standard error (SE). Data with the same letter are not significantly different. Different letters indicate significant differences (p < 0.05) among variants of each fern (lower-case letters) and among the individual ferns of each variant (upper-case letters) according to the Kruskal-Wallis test. Treatment abbreviations: control– 0 mg As·kg^-1^ soil; As1–20 mg As·kg^-1^ soil; As2–100 mg As·kg^-1^ soil. The background soil As content is 16 mg As·kg^-1^ soil. The difference between control and individual As treatments is the spiked As dose plus the 20% As extraction efficiency.

The Asp content was examined in relation to that of Asn in the control and As treatments of all ferns (Fig 9). Differences among ferns in Asp and Asn were found in all treatments. A significant linear correlation was found between As and Asp only for *Ps* (data in S1 Table). Low Asn contents were found in all ferns, though they became elevate with increasing As content (data in S1 Table). Significant differences in Asn between control and As2 treatments were calculated for all studied ferns (Fig 9).

### Effect of CKs homeostasis regulation on controlling the C/N ratio and the content of storage and transport amino acids

Potential relations among the CKs and the other parameters under study are discernible from the graphical presentation of the PCA (Fig 3). These relations were verified using Pearson correlation coefficients for the individual ferns, the significant results of which are summarized in the S2 Table.

The results of the correlation in S2 Table confirmed different CKs homeostasis regulation in the ferns studied. The correlation between ΣCKs and all other groups of CKs was found only for *Ps* (data in S2 Table). In *Pc*-A, a relationship was found between ΣCKs and bCKs, tCKs and sCKs. By contrast, ΣCKs in *Pc*-P correlated only with sCKs, the highest representation in the total CK pool (Table 1, S2 Table). Differences between ferns were found in the correlation of individual CK groups. In *Ps*, all CK groups were significantly correlated (data in S2 Table). On the other hand, in *Pc*-P only a negative correlation was identified between bCKs and ppbCKs. In *Pc*-A, both positive and negative dependences were confirmed between all individual CK groups, especially between bCKs and tCKs as well as the other individual CK groups (data in S2 Table).

The content of CK groups in ferns affected the significant link between C and N assimilation, especially in *Ps* (data in S2 Table). The control of the C/N ratio by CK homeostasis was similar for *Pc*-A and *Ps* ferns, as indicated by the results in S2 Table. In *Pc*-A and *Ps*, positive dependences were confirmed between CK groups, except dCK for *Pc*-A and N assimilation represented by N-NO_3_^-^, N_T_, Gln and Asn. On the other hand, negative dependences were confirmed between CK groups and photosynthesis parameters P_N_ and Fv/Fm (data in S2 Table). These dependences in *Pc*-P were confirmed only for bCKs. In all ferns, only bCKs were positively correlated with N_T_ (data in S2 Table).

## Discussion

The response of the ferns under study to As contamination was assessed based on reduced aboveground biomass, increased *in vivo* concentrations of this element and changes of CK forms and N metabolism in plant aboveground biomass.

As-hyperaccumulators usually enjoy several advantages relative to As-non-hyperaccumulators, including an ability to support rapid growth, efficient root uptake and translocation from roots to shoots [50]. In our experiment, frond biomass declined with increasing As supply in the soil and increasing As content in plants (Figs 1 and 2). Growth reduction is the most common symptom of As stress in plants [51, 52] and represents the result of an activation of plant defense mechanisms and the regulation of stress metabolism.

The current study confirmed a significant difference in ∑CKs–as well as in five functionally different CK groups–between As-hyperaccumulator and As-non-hyperaccumulator ferns. ∑CKs was low in the control treatment of *Ps* relative to *Pc* ferns. A sharp increase in ∑CKs was seen only in the As2 treatment of *Ps*. CK depletion provoked the coordinated activation of As(V) tolerance mechanisms in *Arabidopsis thaliana*, leading to the accumulation of thiol compounds, which are essential for As sequestration [47]. Therefore, according to this finding, CKs form an essential component of plant strategies leading to As(V) tolerance.

Five functionally different CK groups were distinguished according to their structural-physiological roles in the life cycle of plants and during stress conditions. Enhanced accumulation of bCKs was determined in our *Pteris* fern species which differed in As accumulating ability. This phenotypic property of bCK accumulation is a significant condition for overcoming stress. An increase in As content in plants enhanced the accumulation of bCKs in the hyperaccumulator fern *Pteris vittata*, while the opposite trend was found for sensitive plants [44,45]. Stressed plants increased bCK content or regulated the homeostasis of endogenous CK forms. Homeostasis is linked to the development and growth of plants [9–12,17,20,53,54]. In addition to regulation of homeostasis of CKs, the physiological and metabolic processes during the growth and development of plants are affected by the crosstalk in the signal pathway network based on interaction of CKs and auxins [6]. The crosstalk of the growth hormones, i.e. CKs, auxins, gibberellic acid with the stress hormones abscisic acid, salicylic acid, jasmonic acid and ethylene plays an important role in mediating the stress response [3]. Detailed studies on the effect of As on hormonal crosstalk in plants are scant. The effect of As and/or cadmium on auxin/jasmonate interaction was studied in rice roots [55]. Jasmonates interact with auxin, affecting its homeostasis, during the development of roots in the presence of As and/or Cd stress [55]. The crosstalk between the concentrations of indole-3-acetic acid and bCKs was found in non-stressed *Ps* and *Pc*-P, while no significant correlations were determined among growth hormones in the fronds of *Pc*-A [8]. High CKs levels promote auxin biosynthesis in young plant and auxin feeds back on CKs metabolism by inducing CKX [3]. The type of crosstalk (positive or negative) between the hormone signalling pathways determines the defence responses in stressed plants rather than solely the individual contributions of each hormone [6].

The response to leaf senescence or stress by changes in bCK or sCK contents has a direct association with changes in the activity of the enzymes *O*-glucosyltransferases or β-glucosidases. The accumulation of sCKS and dCK in the control variants of ferns was derived enzymatically from bCKs by *O*- or *N*-glucosyltransferases [10,39]. An important finding in our experiment was the accumulation of sCKS and reduction of dCKs in *Pteris* As-hyperaccumulators as well as the increase of both forms in non-hyperaccumulators (Table 1). Long-term As exposure of plants leads to depletion and increased content of *N*-glucosides in dCK form. This finding was confirmed only for As-non-hyperaccumulator *Ps*. *N*-Glucosides are thought to be terminal products of an irreversible deactivation of bCKs [10,39,54]. The accumulation of *N*-glucosides increased during whole-plant development or via a stress effect when there was no final irreversible degradation of bCKs, tCKs and dCKs by CKX located in vacuoles [56,57]. Changes in the accumulation of ppbCKs, tCKs and sCKs represent an important component of the regulation of CKs homeostasis in overcoming stress conditions [10,12,18].

bCKs, tCKs and dCKs are degraded by CKX [10,18,39]. This explains the decline or fluctuation of the contents of the CKs in relation to the toxic effects of As accumulated in ferns (Table 1). Simultaneously, CK degradation by CKX produced isoprene aldehyde, which the plants can utilize in the metabolism of C, for example for the biosynthesis of fatty acids [58]. Conversely, sCKs are not degraded by CKX. The increased content of sCKs provides the source for tCK and bCK reactivation by nonspecific β-glucosidases [17], particularly in *Ps*.

Stress and senescence are known to cause an imbalance in the C/N ratio [59–61]. This negative C/N ratio can be altered by an increased intensity of photosynthesis. Photosynthesis is controlled by changes in phytohormone homeostasis, by increased bCK content or by the regulation of the homeostasis of individual endogenous CK forms [60,62–67]. Depleted plant metabolism is unable to restore the C/N balance by increasing C assimilation. The content of bCKs is subsequently reduced, carbohydrate is remobilized and defoliation is triggered [68].

Chlorophyll fluorescence and net photosynthetic rate were increasingly inhibited with increasing As content (Figs 4 and 5). Our study confirmed the close correlation between these parameters and frond biomass in all ferns (data in S3 Table). These results demonstrated that the reduction of dry biomass reflected inhibition of photosynthesis by As, consistent with the finding that As causes a decline in the plant yield of *Solanum lycopersicum* as well as in Fv/Fm, indicating photosynthetic damage [39]. The negative effects of As on Fv/Fm and the actual quantum efficiency of PSII electron transport previously observed in *P*. *cretica* [69] were not found here. This difference was probably due to the comparative lengths of the experiments (60 days *vs*. 23 weeks).

A decrease in fluorescence below 0.6 for As2 treatments caused a reduction in the C/N ratio. This C/N change induced the enhancement of active CKs, i.e. bCKs and tCK forms. Endogenous bCKs and tCKs play a role in plants’ adaptation to abiotic stress by regulating photosynthetic parameters [42,43] and AA metabolism [24] after interacting with plant receptors [63,64,70]. An increase in bCK content induces the metabolism of stress antioxidants, delay of senescence and overcoming of stress via increased photosynthetic activity via a change in the unfavourable C/N ratio [2,62–67]. Increased bCK delays senescence, and C assimilation in As hyperaccumulators is increased by the enhanced photosynthetic activity [45,46].

The C/N ratio is the result of an interaction of nitrogen metabolism and the intensity of photosynthesis, determining assimilation and carbon metabolism, e.g. via the tricarboxylic acid cycle [68,71–73]. The tricarboxylic acid cycle is linked to amino acid biosynthesis [74], especially to the formation of sAAs and tAAs (mainly Gln and Asn–Figs 8 and 9). Our results confirmed the link between the ratio of C/N and toxic As effects in fronds. Carbon assimilation was reduced and, at the same time, the contents of N-NO_3_^-^ and N_T_ including Asn increased (Figs 6, 7 and 9).

Arsenate seems to disrupt N assimilation, interfering with both the supply of inorganic N to the assimilation pathway and the activity of the pathway itself [28]. By contrast, our results with the control and the As1 treatments were similar and confirmed the considerable accumulation of N_T_ and N-NO_3_^-^, respectively, in As2-treated plants. A minor but not significant increase in nitrate concentration was determined in As-treated *Pityrogramma calomelanos* [33]. The levels of nitrate significantly increased in the roots of both tolerant and sensitive varieties of *Brassica juncea* in response to As treatment, albeit showing an increase only in the shoots of tolerant plants [75]. Changes in N-NO_3_^-^ concentrations were confirmed in the fronds of *P*. *vittata* and *Pteris ensiformis* after seven days of exposure to As [30]. Unlike our results, nitrate content was found to decrease with increasing As concentration [30]. However, the plants were exposed to As for a much shorter period than in the current study. The response of N metabolism to As treatment differs in a variety- and time-dependent manner [30,75]. On the other hand, in another article [76] the effects of As on N metabolism were consistent with our study. We found that increased N uptake by ferns was affected by As, as confirmed by the correlations (S1 Table).

Arsenite-treated plants exhibited elevated concentrations of non-protein ^15^N, which could indicate either the stimulated uptake of nitrate or an interruption of amino acid/protein synthesis [76]. In this article, the activation/deactivation of N metabolism is regarded as a symptom of the alarm phase of stress response in plants, when general metabolic stimulation/inhibition occurs [76]. These changes are time-dependent. Previously published changes in the activation/deactivation of N metabolism are consistent with our results, because the contents of N-NO_3_^-^ and N_T_ (Figs 6 and 7) and Gln and Asn (Figs 8 and 9) increased as a result of As toxicity. Like Cd stress, arsenic-induced stress increased Gln accumulation [49]. The intensity and the duration of the stress affected the changes in sAA and tAA content. Such changes are phenotypically specific [24,35,52]. The accumulation of sAAs and tAAs is associated with the biosynthesis of other AAs [74]. As previously mentioned, increased bCK delays senescence, while C assimilation in As hyperaccumulators is augmented by increased photosynthesis [45,46]. bCKs restored photosynthesis by causing an increase in chlorophyll synthesis [1,2,60,62–67] via the precursor 5-aminolevulonic acid synthesized from Glu [62]. However, if the supply of C assimilated in older leaves is depleted and the difference between the content of C and N is growing, then Asn or Gln accumulate as in our ferns. The ratio of tAAs/sAAs is an indicator of primary nitrogen assimilation in relationship to CK forms.

Changes in N metabolism were reflected in the levels of transport amino acids (Glu and Asp) and their storage amides (Gln and Asn). An increase in Gln was found in As-hypearaccumulating *Pc-*A and *Pc-*P plants as a result of both As treatments. Gln is the major AA used for the storage of amino groups in *Pc-*A and *Pc-*P; however, it also represents a key metabolite, acting as an amino donor to other free AAs, primarily through catalysis by glutamate synthase. This pathway interacts with carbohydrate metabolism and the energy status of plant leaves [77]. Another As-hyperaccumulating fern, *Pityrogramma calomelanos*, was found to tolerate high concentrations of As due to its ability to upregulate the biosynthesis of AAs without substantially disturbing central carbon metabolism [33]. The As-induced increase in Gln levels in our study indicated that AAs biosynthesis was upregulated by As. The Glu/Gln ratio in *Pteris* decreased with increasing content of As in the soil (Table 2). The current study has also shown a higher Glu/Gln ratio in As-non-hyperaccumulator *Ps* compared to both As-hyperaccumulating ferns. The increase in Gln and Glu levels reported in *P*. *calomelanos* suggests that the glutamine synthetase/glutamate synthase cycle is As-responsive [33]. The Glu/Gln ratio is considered an indicator of primary N assimilation, providing information on the balance of NH_4_^+^ and 2-oxoglutarate availability [78].

The highest Asp content was found in the control (*Pc*-A), As1 (*Pc*-P) and As2 (*Ps*) treatments. The amidation of Asp by Gln yields Asn, an amino acid used to store N and to transport it from source to sink. All studied ferns exhibited relatively low Asn contents, which were enhanced with increasing As content in *Pc*-P and *Ps*. Asn accumulates in plants under stress conditions [59]; however, the opposite findings were reported for spinach [52]. As a result of the accumulation of Gln and Asn (Figs 8 and 9), there was a clear lack of assimilated carbon, as Asn and Gln were efficient molecules for the storage of N in organisms [59].

## Conclusions

Our data were helpful in better understanding of toxic As effect on the role of phytohormones that regulate physiological and metabolic processes in plants. Our study confirmed a significant difference in five functionally different CK groups between As-hyperaccumulator and As-non-hyperaccumulator ferns. The significant depletion of C resources in the plant and changes in N metabolism (negative change in C/N ratio) by long-term As stress were confirmed in ferns, especially in *Ps*. The glutamic acid/glutamine ratio, an indicator of primary N assimilation, diminished in all ferns with increased As dose in the soil. The results indicate a large phenotypic diversity of *Pteris* species in As adaptation.

## Supporting information

S1 Fig Arsenic accumulation in fronds of *Pteris cretica* var. Parkerii after 90 days of growth in contaminated soil. Treatment abbreviations: control– 0 mg As·kg^-1^; As1–20 mg As·kg^-1^; As2–100 mg As·kg^-1^; As3–250 mg As·kg^-1^, As4–500 mg As·kg^-1^. The background soil As content is 16 mg As·kg^-1^ soil. The difference between control and individual As treatments is the spiked As dose plus the 20% As extraction efficiency. Experiment was done in year 2016 –data were not published. Data with the same letter are not significantly different. Different letters indicate significant differences among variants according to the Kruskal-Wallis test (p < 0.05).
(TIFF)

S1 Table Statistically significant linear correlation between arsenic and other parameters in individual ferns. Parameter abbreviations: DW–yield of dry frond biomass; As–arsenic; net photosynthetic rate; Asp–aspartic acid; Asn–asparagine; Glu–glutamic acid; Gln–glutamine; N_T_−total nitrogen; N-NO_3_^-^ –nitrate nitrogen; Fv/Fm–chlorophyll fluorescence; ΣCKs–total cytokinins; bCKs–bioactive cytokinin forms; dCKs–inactive (or weakly active) cytokinin forms; tCKs–transport cytokinin forms; sCKs–storage cytokinin forms; ppbCKs–primary products of cytokinin biosynthesis. “-” ─ correlation was not statistically significant.
(DOCX)

S2 Table Statistically significant linear correlation between cytokinins groups and selected parameters in individual ferns. Parameter abbreviations: DW–yield of dry frond biomass; net photosynthetic rate; Asp–aspartic acid; Asn–asparagine; Glu–glutamic acid; Gln–glutamine; N_T_−total nitrogen; N-NO_3_^-^ –nitrate nitrogen; Fv/Fm–chlorophyll fluorescence; ΣCKs–total cytokinins; bCKs–bioactive cytokinin forms; dCKs–inactive (or weakly active) cytokinin forms; tCKs–transport cytokinin forms; sCKs–storage cytokinin forms; ppbCKs–primary products of cytokinin biosynthesis. “-” ─ correlation was not statistically significant.
(DOCX)

S3 Table Statistically significant linear correlation between dry frond biomass and parameters of photosynthesis in individual ferns. Parameter abbreviations: DW–yield of dry frond biomass; P_N_−net photosynthetic rate; Fv/Fm–chlorophyll fluorescence.
(DOCX)
