# Supplementary figures and images for: Response of cytokinins and nitrogen metabolism in the fronds of Pteris sp. under arsenic stress
Source: PLoS One. 2020 May 15;15(5):e0233055. doi: 10.1371/journal.pone.0233055 (PMC7228123; doi:10.1371/journal.pone.0233055)

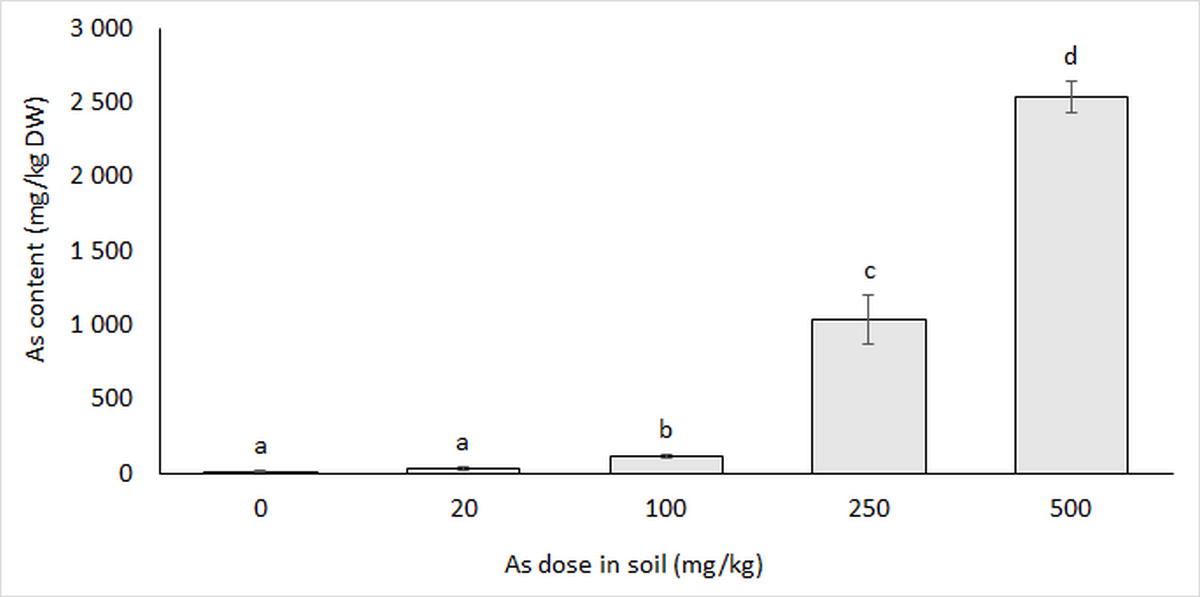

Supplement: S1 Fig — Treatment abbreviations: control– 0 mg As·kg-1; As1–20 mg As·kg-1; As2–100 mg As·kg-1; As3–250 mg As·kg-1, As4–500 mg As·kg-1. The background soil As content is 16 mg As·kg-1 soil. The difference between control and individual As treatments is the spiked As dose plus the 20% As extraction efficiency. Experiment was done in year 2016 –data were not published. Data with the same letter are not significantly different. Different letters indicate significant differences among variants according to the Kruskal-Wallis test (p < 0.05). (TIFF) [file pone.0233055.s001.tiff]
