# Supplementary material for: Response of cytokinins and nitrogen metabolism in the fronds of Pteris sp. under arsenic stress
Source: PLoS One. 2020 May 15;15(5):e0233055. doi: 10.1371/journal.pone.0233055 (PMC7228123; doi:10.1371/journal.pone.0233055)
Supplement: S2 Table — Parameter abbreviations: DW–yield of dry frond biomass; net photosynthetic rate; Asp–aspartic acid; Asn–asparagine; Glu–glutamic acid; Gln–glutamine; NT−total nitrogen; N-NO3- –nitrate nitrogen; Fv/Fm–chlorophyll fluorescence; ΣCKs–total cytokinins; bCKs–bioactive cytokinin forms; dCKs–inactive (or weakly active) cytokinin forms; tCKs–transport cytokinin forms; sCKs–storage cytokinin forms; ppbCKs–primary products of cytokinin biosynthesis. “-” ─ correlation was not statistically significant. (DOCX) [file pone.0233055.s003.docx]

**S2 Table.** **Statistically significant linear correlation between cytokinin groups and selected parameters in individual ferns.** Parameter abbreviations: DW – yield of dry frond biomass; P_N_ – net photosynthetic rate; Asp – aspartic acid; Asn – asparagine; Glu – glutamic acid; Gln – glutamine; N_T_ – total nitrogen; N-NO_3_^-^ – nitrate nitrogen; Fv/Fm – chlorophyll fluorescence; ΣCKs – total cytokinins; bCKs – bioactive cytokinin forms; dCKs – inactive (or weakly active) cytokinin forms; tCKs – transport cytokinin forms; sCKs – storage cytokinin forms; ppbCKs – primary products of cytokinin biosynthesis. “-” ─ correlation was not statistically significant.

|  | *P. cretica* - Albo-lineata | | *P. cretica* - Parkerii | | *P. straminea* | |
| --- | --- | --- | --- | --- | --- | --- |
| ΣCKs | r | *p* | r | *p* | r | *p* |
| bCKs | 0.75 | 0.019 | - | - | 0.99 | 0.000 |
| tCKs | 0.90 | 0.001 | - | - | 0.98 | 0.000 |
| dCKs | - | - | - | - | 0.99 | 0.000 |
| sCKs | 0.98 | 0.000 | 0.99 | 0.000 | 0.99 | 0.000 |
| ppbCKs | - | - | - | - | 0.89 | 0.001 |
| DW | - 0.91 | 0.001 | - | - | - 0.99 | 0.000 |
| N_T_ | 0.88 | 0.002 | - | - | 0.98 | 0.000 |
| N-NO_3_^-^ | 0.83 | 0.005 | - | - | 0.99 | 0.000 |
| P_N_ | -0.97 | 0.000 | - | - | -0.78 | 0.012 |
| Fv/Fm | -0.95 | 0.000 | - | - | -0.86 | 0.003 |
| Glu | - | - | 0.83 | 0.000 | 0.78 | 0.014 |
| Gln | 0.84 | 0.005 | - | - | 0.95 | 0.000 |
| Asp | - | - | 0.94 | 0.000 | 0.98 | 0.000 |
| Asn | 0.76 | 0.017 | - | - | 0.97 | 0.000 |
| bCKs | r | *p* | r | *p* | r | *p* |
| tCKs | 0.94 | 0.000 | - | - | 0.99 | 0.000 |
| dCKs | - 0.77 | 0.016 | - | - | 0.96 | 0.000 |
| sCKs | - | - | - | - | 0.97 | 0.000 |
| ppbCKs | 0.84 | 0.004 | - 0.97 | 0.000 | 0.92 | 0.000 |
| DW | - 0.95 | 0.000 | - 0.69 | 0.041 | - 0.98 | 0.000 |
| N_T_ | 0.97 | 0.000 | 0.67 | 0.048 | 0.99 | 0.000 |
| N-NO_3_^-^ | 0.98 | 0.000 | 0.96 | 0.000 | 0.99 | 0.000 |
| P_N_ | - | - | -0.97 | 0.000 | -0.84 | 0.004 |
| Fv/Fm | -0.72 | 0.030 | - | - | -0.87 | 0.002 |
| Glu | 0.71 | 0.033 | -0.84 | 0.005 | 0.80 | 0.010 |
| Gln | 0.87 | 0.002 | 0.67 | 0.049 | 0.97 | 0.000 |
| Asp | - | - | - | - | 0.99 | 0.000 |
| Asn | 0.97 | 0.000 | 0.97 | 0.000 | 0.99 | 0.000 |
| tCKs | r | *p* | r | *p* | r | *p* |
| dCKs | - 0.73 | 0.025 | - | - | 0.95 | 0.000 |
| sCKs | 0.81 | 0.008 | - | - | 0.95 | 0.000 |
| ppbCKs | 0.79 | 0.010 | - | - | 0.95 | 0.000 |
| DW | - 0.99 | 0.000 | - | - | - 0.96 | 0.000 |
| N_T_ | 0.99 | 0.000 | - | - | 0.98 | 0.000 |
| N-NO_3_^-^ | 0.99 | 0.000 | - | - | 0.98 | 0.000 |
| P_N_ | -0.78 | 0.012 | - | - | -0.87 | 0.002 |
| Fv/Fm | -0.90 | 0.001 | - | - | -0.87 | 0.002 |
| Glu | - | - | - | - | 0.84 | 0.004 |
| Gln | 0.94 | 0.000 | - | - | 0.98 | 0.000 |
| Asp | - | - | - | - | 0.99 | 0.000 |
| Asn | 0.96 | 0.000 | - | - | 0.99 | 0.000 |
| dCKs | r | *p* | r | *p* | r | *p* |
| sCKs | - | - | - | - | 0.99 | 0.000 |
| ppbCKs | - 0.97 | 0.000 | - | - | 0.81 | 0.008 |
| DW | 0.68 | 0.044 | - | - | - 0.99 | 0.000 |
| N_T_ | -0.71 | 0.031 | -0.70 | 0.038 | 0.99 | 0.000 |
| N-NO_3_^-^ | -0.78 | 0.013 | - | - | 0.99 | 0.000 |
| P_N_ | - | - | - | - | -0.68 | 0.043 |
| Fv/Fm | - | - | 0.71 | 0.032 | -0.77 | 0.015 |
| Glu | -0.88 | 0.002 | - | - | 0.72 | 0.028 |
| Gln | -0.71 | 0.032 | - | - | 0.93 | 0.000 |
| Asp | -0.73 | 0.025 | - | - | 0.95 | 0.000 |
| Asn | -0.85 | 0.004 | - | - | 0.93 | 0.000 |
| sCKs | r | *p* | r | *p* | r | *p* |
| ppbCKs | - | - | - | - | 0.85 | 0.004 |
| DW | - 0.82 | 0.006 | - | - | - 0.98 | 0.000 |
| N_T_ | 0.78 | 0.012 | - | - | 0.97 | 0.000 |
| N-NO_3_^-^ | 0.72 | 0.028 | - | - | 0.98 | 0.000 |
| P_N_ | -0.99 | 0.000 | - | - | -0.73 | 0.025 |
| Fv/Fm | -0.92 | 0.000 | - | - | -0.83 | 0.006 |
| Glu | - | - | 0.85 | 0.003 | 0.74 | 0.024 |
| Gln | 0.75 | 0.019 | - | - | 0.91 | 0.001 |
| Asp | - | - | 0.97 | 0.000 | 0.95 | 0.000 |
| Asn | - | - | - | - | 0.94 | 0.000 |
| ppbCKs | r | *p* | r | *p* | r | *p* |
| DW | - 0.76 | 0.018 | 0.73 | 0.025 | - 0.84 | 0.005 |
| N_T_ | 0.78 | 0.013 | -0.73 | 0.026 | 0.87 | 0.002 |
| N-NO_3_^-^ | 0.85 | 0.004 | -0.99 | 0.000 | 0.87 | 0.002 |
| P_N_ | - | - | 0.99 | 0.000 | -0.97 | 0.000 |
| Fv/Fm | - | - | - | - | -0.95 | 0.000 |
| Glu | 0.86 | 0.003 | 0.83 | 0.005 | 0.87 | 0.002 |
| Gln | 0.76 | 0.017 | -0.68 | 0.043 | 0.89 | 0.001 |
| Asp | 0.69 | 0.041 | - | - | 0.94 | 0.000 |
| Asn | 0.90 | 0.001 | -0.99 | 0.000 | 0.95 | 0.000 |
