# Supplementary material for: Response of cytokinins and nitrogen metabolism in the fronds of Pteris sp. under arsenic stress
Source: PLoS One. 2020 May 15;15(5):e0233055. doi: 10.1371/journal.pone.0233055 (PMC7228123; doi:10.1371/journal.pone.0233055)
Supplement: S3 Table — Parameter abbreviations: DW–yield of dry frond biomass; PN−net photosynthetic rate; Fv/Fm–chlorophyll fluorescence. (DOCX) [file pone.0233055.s004.docx]

**S3 Table.** **Statistically significant linear correlation between dry frond biomass and parameters of photosynthesis in individual ferns.** Parameter abbreviations: DW – yield of dry frond biomass; P_N_ – net photosynthetic rate; Fv/Fm – chlorophyll fluorescence.

| Parameters | *P. cretica* - Albo-lineata | | *P. cretica* - Parkerii | | *P. straminea* | |
| --- | --- | --- | --- | --- | --- | --- |
| DW | r | *p* | r | *p* | r | *p* |
| P_N_ | 0.81 | 0.008 | 0.80 | 0.010 | 0.72 | 0.028 |
| Fv/Fm | 0.88 | 0.002 | 0.99 | 0.000 | 0.79 | 0.012 |
